# *Nypa fruticans* Wurmb. Vinegar’s Aqueous Extract Stimulates Insulin Secretion and Exerts Hepatoprotective Effect on STZ-Induced Diabetic Rats

**DOI:** 10.3390/nu9090925

**Published:** 2017-08-23

**Authors:** Nor Adlin Yusoff, Vuanghao Lim, Bassel Al-Hindi, Khairul Niza Abdul Razak, Tri Widyawati, Dwi Rita Anggraini, Mariam Ahmad, Mohd Zaini Asmawi

**Affiliations:** 1 Integrative Medicine Cluster, Advanced Medical and Dental Institute, Universiti Sains Malaysia, Bertam, Kepala Batas, Penang 13200, Malaysia; vlim@usm.my; 2 School of Pharmaceutical Sciences, Universiti Sains Malaysia, Penang 11800, Malaysia; basselalhindi@yahoo.com (B.A.-H.); niza@usm.my (K.N.A.R.); mariam@usm.my (M.A.); amzaini@usm.my (M.Z.A.); 3 Pharmacology and Therapeutic Department, Medical Faculty, Universitas Sumatera Utara, Medan 20155, Indonesia; tri.widyawati@usu.ac.id; 4 Anatomy Department, Medical Faculty, Universitas Sumatera Utara, Medan 20155, Indonesia; dwirita@usu.ac.id

**Keywords:** antihyperglycaemic, hepatoprotective, *Nypa fruticans* Wurmb., vinegar, diabetes, RIN-5F, natural product

## Abstract

Background: An aqueous extract (AE) of vinegar made from *Nypa fruticans* Wurmb. can improve postprandial glucose levels in normoglycaemic rats. The aim of this study was to evaluate its antihyperglycaemic activity further using in vivo and in vitro approaches. Methods: AE was administered to streptozotocin (STZ)-induced diabetic rats twice daily at three doses (1000, 500, and 250 mg/kg b.w.) for 12 days p.o. Several biochemical analyses and a histological study of the pancreas and liver were performed, accompanied by a cell culture assay. Results: As compared to diabetic control (DC), AE at the doses of 500 and 1000 mg/kg b.w. caused significant reduction (*p* < 0.05) of blood glucose, total cholesterol and triglycerides levels, with positive improvement of serum insulin levels. Interestingly, immunohistochemical staining of the pancreas suggested no β-cell regeneration, despite significant increase in insulin production. AE-treated groups, however, showed overall restoration of the hepatic histoarchitecture of STZ-induced liver damage, suggesting a possible hepatoprotective effect. The pancreatic effect of AE was further studied through RIN-5F cell culture, which revealed a positive stimulatory effect on insulin release at a basal glucose concentration (1.1 mM). Conclusion: *Nypa fruticans* Wurmb. vinegar’s aqueous extract exerts its antihyperglycaemic activity, at least in part, through insulin stimulatory and hepatoprotective effects.

## 1. Introduction

Functional foods have captured considerable interest as potential alternative therapies for the treatment of diabetes mellitus (DM) and its complications [1,2,3,4]. Incorporating functional foods in the dietary regimen of diabetic patients proved to favourably influence the blood glucose levels of such patients [5,6,7]. According to the Functional Food Center, USA, functional foods are defined as natural or processed foods that contain known or unknown biologically active compounds which in defined, effective and nontoxic amounts provide a clinically proven and documented health benefit for the prevention, management, or treatment of a chronic disease [8]. Besides having minimal side effects in clinical practices and being obtainable at a relatively low cost, functional foods have garnered further attention due to the increase in scientific studies regarding their effectiveness.

Vinegar is one of the widely consumed functional foods worldwide. The consumption of vinegar with meals was used as a home remedy for diabetes before the advent of pharmacological glucose-lowering drugs [9]. *Nypa fruticans* Wurmb. vinegar locally known as nipa palm vinegar (NPV) is one of the traditional vinegars produced by fermentation of nira, the sap of the nipa palm (*Nypa fruticans* Wurmb.). It is predominantly consumed by populations in East Asia. In our previous study, an aqueous extract (AE) of NPV showed the strongest blood glucose lowering activity among several tested NPV extracts [10]. In another investigation dealing with the mechanisms of the antihyperglycaemic action of AE, a significant effect was revealed in terms of alleviating postprandial hyperglycaemia in normolgycaemic rats [11].

The liver and the pancreas are two vital regulatory organs for glucose homeostasis. Abnormalities in both are strongly associated with the prevalence and progression of diabetes mellitus [12,13,14]. Several researches have demonstrated the positive effect of vinegars (i.e., balsamic, apple cider, and pineapple) in stimulating insulin secretion, improving the function of pancreatic β cells [15], and protecting the liver cells [16,17]. Hence, the present study was designed to evaluate the possible effects of *Nypa fruticans* Wurmb. vinegar’s aqueous extract on the pancreas and the liver using in vivo and in vitro approaches.

## 2. Materials and Methods

### 2.1. Chemicals

D-Glucose and streptozotocin (STZ) were purchased from Sigma Aldrich (St. Louis, MO, USA). Insulin ELISA kit was obtained from Crystal Chem, Inc. (Downers Grove, IL, USA). Metformin (Glucophage^®^) was purchased from Bristol-Myers Squibb Co. (New York, NY, USA). All other chemicals and solvents used were of reagent grade.

### 2.2. Vinegar Preparation and Extraction

NPV used in the study was supplied by a local producer from Titi Bakong, Yan, Kedah, Malaysia (5°48′9.42′′ N, 100°22′35.32′′ E). Several parts of nipa palm were authenticated and voucher specimens (USM.Herbarium 11541) were deposited at the Herbarium Unit, School of Biological Sciences, Universiti Sains Malaysia (USM). NPV was extracted using a liquid-liquid extraction method as described by Qiu et al. [18] with minor modifications. NPV (500 mL) was concentrated to a final volume of 250 mL at 37 °C using a vacuum rotary evaporator (Buchi Labortechnik, AG CH-9230, Flawil, Switzerland). Concentrated NPV was first extracted with ethyl acetate at a ratio of 1:1. The ethyl acetate layer (upper layer) was then separated; the residue was collected and considered as the aqueous layer. The aqueous layer was concentrated under reduced pressure at 40 °C using a vacuum rotary evaporator; and was lyophilized using a freeze-drier (Labconco Corporation, Kansas, MO, USA) to yield AE (yield 85%). AE was stored in the freezer at −4 °C until further use.

### 2.3. Experimental Animals

**Figure 1:**
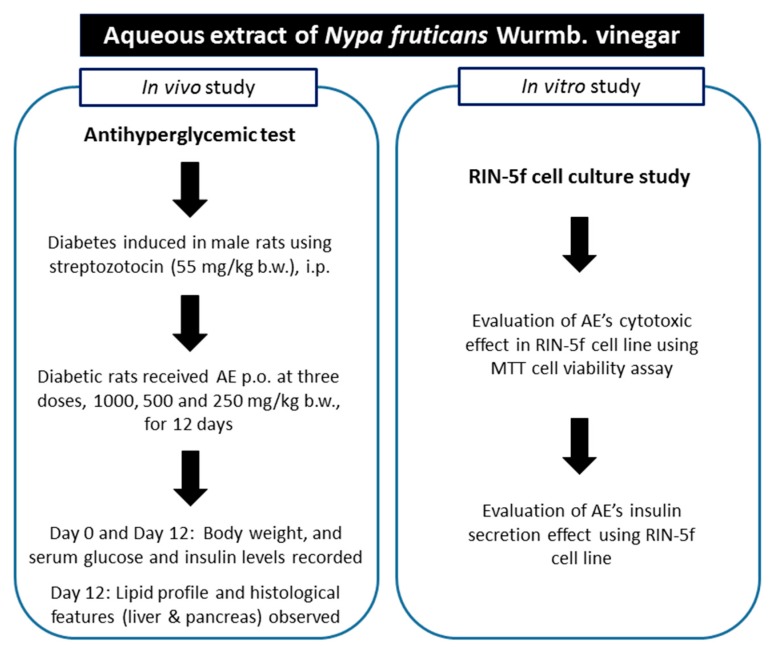
Overview of the experimental design.

Adult male Sprague-Dawley rats weighing 200 to 250 g were obtained from the Animal Research and Service Centre, Universiti Sains Malaysia (USM). All animals were housed and acclimatized in a well-ventilated animal transit room (12 h light/12 h dark cycle) at the School of Pharmaceutical Sciences, USM. Throughout the experiment, the rats had ad libitum access to water and food pellets (Gold Coin Feedmills, Butterworth, Malaysia). The study protocol (depicted in Figure 1) was approved by the Animal Ethic Committee at USM (Approval number: USM/Animal Ethics Approval/2011/(71) (326)). Diabetes was induced chemically in rats using STZ via the intraperitoneal (i.p.) route at a dose of 55 mg/kg b.w. (body weight). Three days after injection, the animals whose fasting blood glucose levels were within the range of 11–20 mmol/L were selected for the study.

### 2.4. Antihyperglycaemic Test of Different Doses of AE

A total of 36 normal rats were used and randomly divided into 6 groups of 6 rats each. The assigned groups were as follows; Group 1: Normal control (NC) + distilled water (10 mL/kg b.w), Group II: Diabetic control (DC) + distilled water (10 mL/kg b.w.), Group III: Diabetic + Metformin (500 mg/kg b.w.), Group IV: Diabetic + AE (1000 mg/kg b.w.), Group V: Diabetic + AE (500 mg/kg b.w.) and Group VI: Diabetic + AE (250 mg/kg b.w.). The oral treatments were given twice daily, with a 12-h dosing interval for a period of 12 days. Blood glucose levels were recorded on days 0, 3, 6, 9 and 12 following an overnight fast. At the end of the study the rats were euthanized, and blood samples were collected via cardiac puncture for insulin and lipid profile analyses. An insulin analysis was performed using an ELISA kit (Ultra Sensitive Rat Insulin, Crystal Chem, Inc., Downers Grove, IL, USA), whereas the lipid profile was estimated by enzymatic kinetic methods using an automated chemistry analyser (Siemens ADVIA 2400, Siemens Healthcare, Germany. The pancreas and the liver were excised for histological studies.

### 2.5. Pancreatic Immunohistopathological Study

The pancreatic tissues were fixed in 10% buffered formalin, processed using a tissue processor, Citadel 1000 histokinette (Shandon Scientific Ltd., Cheshire, UK) and embedded in paraffin using Histo-Center II-N (Barnstead/Thermolyne Corp., Dubuque, IA, USA). The paraffin-embedded tissues were sectioned into 5-μm slices and mounted on a poly-l-lysine-coated microscope slide. Immunohistochemical staining was performed using guinea-pig polyclonal antibody of rat insulin (DAKO, Glostrup, Denmark). The sections were deparaffinised. Endogenous peroxidises were quenched by treatment with 3% hydrogen peroxide in methanol, followed by washing in phosphate buffered saline (PBS). Then, the sections were incubated in a diluted normal serum for 20 min to block the non-specific binding of immunoglobulin G lgG. The primary antibody, guinea pig polyclonal insulin antibody (DAKO, Glostrup, Denmark) was applied for 30 min, followed by washing with PBS for 5 min. The sections were incubated for 30 min with the 1`biotinylated secondary antibody, followed by washing. Following a further 30-min incubation period in a Vectastain ABC kit (Vector Laboratories, Burlingame, CA, USA), washing was performed for 5 min before diaminobenzidine (DAB) (DAKO, Glostrup, Denmark was added for 3–5 min. The sections were lightly counterstained with Harris Haematoxylin, dehydrated, cleared and mounted. The islets of Langerhans were examined under a light microscope (Leica^®^ DMi1, Leica Microsystems, Wetzlar, Hesse, Germany). The digital images were analysed using an image analyser (Leica^®^ microsystem Qwin plus) to allow the calculation of the insulin-containing area of β cells (%) over the total area of the Langerhans islet.

### 2.6. Histological Study of Liver

Liver tissues were fixed, processed and embedded as mentioned in Section 2.5. Then, paraffin embedded 5-μm tissue sections were cut using a Reichert-Jung Histocut 820 II microtome (Cambridge Instrument GmbH, Nussloch, Germany). The sections were stained with Haematoxylin and Eosin (H & E) [19].

### 2.7. RIN-5F Cell Culture

The RIN-5F β cell line was purchased from ATCC, Mannasas, USA (ATCC^®^CRL2058™). The cell line was maintained in an RPMI 1640 medium containing 4.5 g/L d-glucose, 1.5 g/L sodium bicarbonate, 1 mM sodium pyruvate, 10 mM HEPES and 300 mg/L l-glutamine (Gibco A10491-01). The medium was supplemented with 10% foetal bovine serum (FBS) and 1% penicillin-streptomycin mixtures. The cell line was incubated at 37 °C with 5% CO_2_.

#### 2.7.1. Cytotoxicity Assay

The cytotoxic effect of AE on RIN-5F cells was analysed using an MTT (MTT: 3-(4,5-dimethylthiazol-2-yl)-2,5-diphenyltetrazoliumbromide) assay by measuring the reduction of MTT to formazan [20]. The cells were seeded in a 96-well plate at 1 × 10^4^ cells/well and incubated in a CO_2_ incubator at 37 °C for 72 h. They were exposed to AE at the following concentrations of 100, 50, 25, 12.5, 6.25 and 3.125 μg/mL for 24 h. After a 24-h exposure, the medium was removed and washed with PBS. Twenty microliters of the MTT solution (5 mg/mL) were added to each well and the cells were incubated in the dark for 4 h more. Thereafter, the medium was removed, and the formazan crystal was dissolved with 100 μL DMSO for 1 h. Lastly, absorbance was measured at 570 nm using a microplate reader.

#### 2.7.2. Insulin Assay

Insulin secretion was measured as previously described by Hassan et al. [21] with minor modifications. The cells were seeded in a 24-well plate at a density of 4 × 10^5^ cells/well and incubated in a CO_2_ incubator at 37 °C. The medium was removed after 72 h; and traces of the culture medium were washed off with PBS (3 times). The cells were pre-incubated for 40 min at 37 °C with 1 mL of the Krebs Ringer Bicarbonate (KRB) buffer, containing 115 mM NaCl, 4.7 mM KCl, 1.28 mM CaCl_2_, 1.2 mM Mg_2_SO_4_, 1.2 mM KH_2_PO_4_, 10 mM HEPES and 24 mM NaHCO_3_ (pH 7.4). The buffer was then replaced with 1 mL KRB buffer solution supplemented with 1.1 mM glucose in the absence/presence of AE (25, 12.5 or 6.25 µg/mL) and glibenclamide (0.988 μg/mL, 9.88 μg/mL and 98.8 μg/mL). The test was performed by incubating the cells for 30 min at 37 °C. Following incubation, aliquots were collected from each well for an insulin assay conducted using an ELISA kit according to the manufacturer’s protocol (Cloud-Clone Corp., Katy, TX, USA).

### 2.8. Statistical Analysis

Data was expressed as the mean ± standard error of the mean (S.E.M). The results were analysed using one-way analysis of variance (ANOVA) followed by Dunnett’s comparison test. The results were statistically significant at *p* < 0.05.

## 3. Results

### 3.1. Effect of Repeated Oral Administration of AE on Fasting Blood Glucose, Serum Insulin and Body Weight

The effects observed after the 12-day twice-daily oral administration of AE on the levels of blood glucose, serum insulin and body weight in STZ-induced diabetic rats are presented in Table 1. Administered at 250, 500, and 1000 mg/kg b.w., AE showed a dose-dependent effect as maximum blood glucose reduction was observed in the diabetic rats treated with the highest dose (1000 mg/kg b.w.), followed by those treated with 500 mg/kg b.w. The reduction in blood glucose for the two doses was at 49.2% and 56.6%, respectively. AE at the dose of 250 mg/kg b.w. failed to normalize the blood glucose levels of diabetic rats after 12 days of treatment. Metformin, given at the dose of 500 mg/kg b.w., lowered blood glucose levels by 47.0% and 57.9% at the end of study, as compared with the respective initial value (day 0) and the value for DC on day 12, respectively.

Distinct from its effect on blood glucose levels, STZ caused significant decreases in serum insulin levels as shown when comparing DC with NC on day 0 (48.6%; *p* < 0.001). The administration of AE at three doses did not cause any significant increase in the serum insulin levels of diabetic treated rats, as compared with DC. Compared with day 0, 500 mg/kg b.w. of AE, however, recorded significant improvement in the insulin level on day 12 (13.6%, *p* < 0.05).

As predicted, DC rats recorded a significant loss of body weight compared to its initial value and the value of NC on day 12 (*p* < 0.01 and *p* < 0.001, respectively). The metformin-treated group only experienced mild and insignificant weight loss by the end of the study (day 12). AE-treated groups were comparable to DC as well in terms of body weight by the end of the study with no significant loss or gain recorded.

### 3.2. Effect on Lipid Profile

Table 2 shows the serum levels of total cholesterol (TC), triglycerides (TG), high density lipoprotein (HDL) cholesterol, low density lipoprotein (LDL) cholesterol and the atherogenic index of plasma (AIP) of normal and STZ-induced diabetic rats. As compared with NC, significantly increased TG levels (34.93%, *p* < 0.05) in DC were not accompanied by significant changes in the levels of TC, HDL or LDL cholesterol. The administration of metformin to diabetic rats over the course of 12 days did not cause any significant changes in the serum lipid profile as compared to DC. On the other hand, AE, at the dose of 1000mg/kg b.w., significantly decreased the levels of TG (50.28%, *p* < 0.01), with no significant changes in the levels of TC and HDL cholesterol as compared to DC. The doses of 500 and 250 mg/kg b.w. of AE showed comparable effects on the lipid profile; TC and TG levels were significantly decreased (38.96% and 38.96%, *p* < 0.05; 58.95% and 43.86%, *p* < 0.05), and so was HDL cholesterol (38.41% and 42.92%, *p* < 0.05). AIP indicated a lower risk of cardiovascular disease in the diabetic rats treated with AE at the doses of 1000 mg/kg b.w. and 500 mg/kg b.w. (AIP values were 0.07 and 0.1, respectively).

### 3.3. Immunohistochemical Study of Insulin-Containing β-Cells of the Pancreas

**Figure 2:**
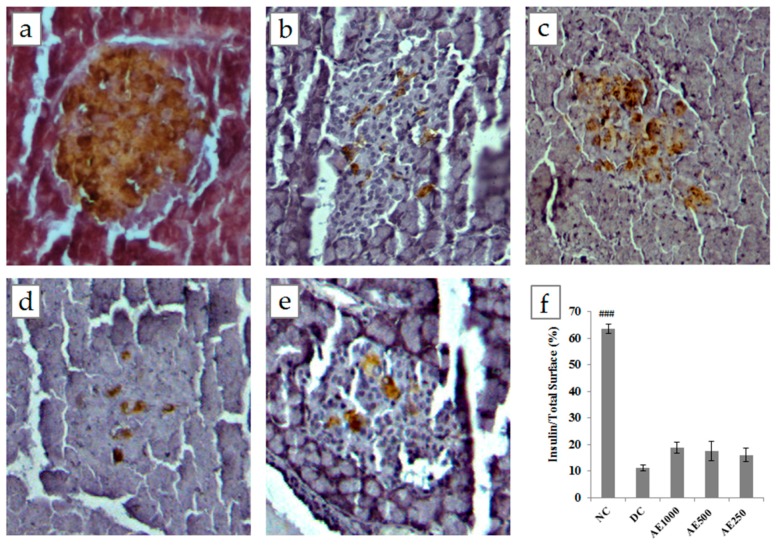
Immunohistochemistry staining of pancreatic β cells after 12 days, twice daily oral treatment. (**a**) NC—intact Langerhans islets with insulin-containing area (dark brown) occupying mainly in the central zone; (**b**) DC—Langerhans islets appeared to be shrunk with significant reduction in the insulin-containing area; (**c**–**e**) AE (1000 mg/kg b.w., 500 mg/kg b.w., 250 mg/kg b.w., respectively)—Langerhans islets appeared to be distorted and smaller. These groups showed larger insulin-expressing areas than those in DC but were statistically insignificant; (**f**) The percentage of insulin-expressing areas in pancreatic islets. Values were expressed as mean ± S.E.M (*n* = 10); ### *p* < 0.001 versus DC.

Insulin-containing β cells refer to viable β-cells capable of secreting insulin, and are represented by dark brown granules. As can be seen in Figure 2, in NC, the islets of Langerhans showed a normal histological structure with insulin-expressing areas occupying mainly the central zone. In contrast, the islets of Langerhans in DC appeared to be shrunken and irregular in shape. All groups treated with AE at the doses of 1000, 500, and 250 mg/kg b.w. were observed to have distorted and smaller pancreatic islets as compared with NC. These groups, however, showed larger insulin-expression areas than those of DC, though the difference was statistically insignificant.

### 3.4. Hepatoprotective Effect of AE

**Figure 3:**
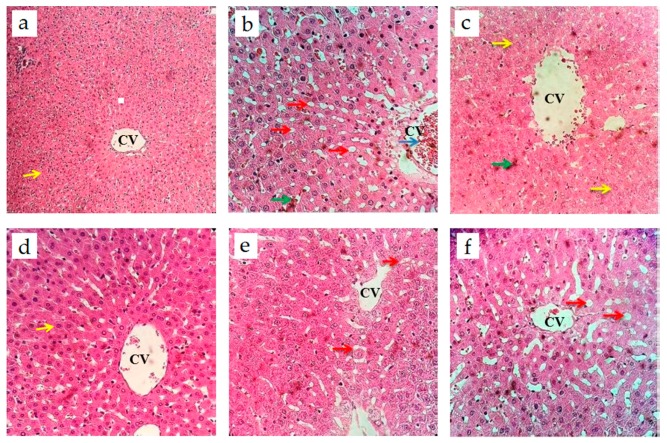
Histology of the livers of experimental rats after 12 days of twice-daily oral treatment with AE: (**a**) NC—the hepatic lobular cells and architecture seemed to be normal; (**b**) DC—Clear red blood cell congestion in the portal vein system (blue arrow) and sinusoids (green arrow), with the accumulation of micro droplets of fat in the centrilobular portion (red arrows); (**c**) Metformin (500 mg/kg b.w.)—Hepatocyte swelling (yellow arrow) and sinusoidal congestion (green arrow) with a decreased number of micro droplets of fat deposit; (**d**) AE (1000 mg/kg b.w.)—normal liver morphology with normal hepatocytes and a central vein; (**e**) AE (500 mg/kg b.w.)—decreased microvesicular fatty change (red arrow); (**f**) AE (250 mg/kg b.w.)—decreased fat micro droplets (red arrow). CV, central vein.

The histological study of the liver revealed significant changes in the general histological organisation of the hepatic tissue between NC and DC (Figure 3). In NC, the hepatic lobular structure seemed to be normal with no red blood cell congestion in the central vein and the sinusoids. The polygonal hepatocytes appeared to be having single or polynuclei; no fat deposits in the centrilobular portions of the livers were detected. On the other hand, DC showed a red blood cell congestion in the portal vein system and the sinusoids, with the accumulation of micro droplets of fat in the centrilobular portion. Treatment with AE at the dose of 1000 mg/kg b.w. restored the histological appearance of the liver as hepatocytes and the central vein appeared to be normal. Meanwhile, the doses of 500 and 250 mg/kg b.w. resulted in better improvement of the liver histology as no congestion of red blood cells was noted in the central vein and sinusoids, as compared with DC. However, an accumulation of micro droplets of fat was still found with decrease amount.

### 3.5. Cytotoxicity Effect of AE on RIN-5F Cells

AE at the concentrations ranging from 3.125 μg/mL to 100 μg/mL did not cause any cytotoxic effect to RIN-5F cells, indicating that the effect of AE on insulin secretion was not due to its cytotoxicity.

### 3.6. Insulin Secretion Effect of AE on RIN-5F Cells

**Figure 4:**
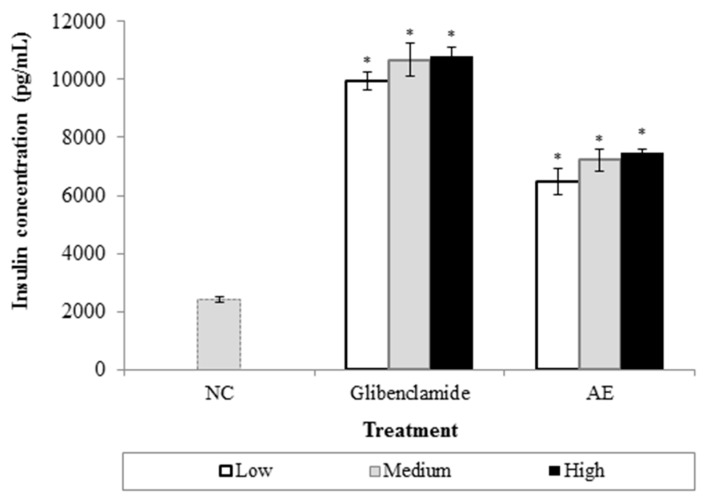
Effects of AE on insulin secretion by RIN-5F cells. Values are expressed as mean ± S.E.M (*n* = 6). The concentrations of glibenclamide were determined as follows: low (0.988 µg/mL); medium (9.88 µg/mL), and; high (98.8 µg/mL); whereas for AE, the range was as follows: low (6.25 µg/mL); medium (12.5 µg/mL), and; high (25 µg/mL). * *p* < 0.05 is significant as compared to NC.

As shown in Figure 4, glibenclamide (0.988–98.8 µg/mL) significantly stimulated the release of insulin from RIN-5F cells at the basal glucose concentration of 1.1 mM. At the concentrations of 6.25, 12.5 and 25 µg/mL, AE produced similar stimulatory effects as glibenclamide, albeit rather weaker. No significant difference was seen in the stimulatory effect for those three applied doses.

## 4. Discussion

This study was conducted to evaluate the antihyperglycaemic effect of 12-day oral administration of AE (1000, 500, and 250 mg/kg b.w.) on STZ-induced diabetic rats. Further investigations focusing on the pancreas and liver were carried out in an attempt to understand the possible effect of AE on these two vital organs that are involved in glucose homeostasis. From the data presented in Table 1, it can be concluded that AE exerted a dose-dependent effect on serum glucose levels as the maximum reduction of blood glucose was observed in the diabetic rats treated with the highest dose (1000 mg/kg b.w.), followed by those treated with 500 and 250 mg/kg b.w. Furthermore, AE-treated groups showed no significant differences in terms of weight loss, as compared to DC. Body weight loss which was observed in the untreated and treated diabetic rats might happen due to increased muscle wasting and loss of protein from tissues [22]. This finding is in agreement with Soltan and Shehata [23], as they reported no significant difference between the weights of rats treated with various types of vinegars as compared with DC. Moon et al. [24] studied the effect of persimmon vinegar on weight gain and reported the same outcome.

Hypercholesterolemia and hypertriglyceridemia are primary factors involved in the development of atherosclerosis and coronary heart disease—two of the secondary complications of diabetes [25,26]. STZ significantly increased TG levels with no significant changes in TC, HDL cholesterol and LDL cholesterol as compared with the levels shown in normal rats (Table 2). AE at the dose of 1000 mg/kg b.w. showed significant reduction in serum TG, but it did not ameliorate the levels of TC. Then again, as the dose was decreased to 500 and 250 mg/kg b.w., TG and TC levels were reduced. This finding could imply that lower AE concentrations produce better effects on the lipid profile. Taken together, the findings suggest that the observed hypolipidemic effect is not mediated by better glycaemic control.

Hypoinsulinaemia, one of the major characteristics of STZ-induced diabetic rats, occurs due to irreversible destruction of insulin-producing β cells in the pancreatic islets of Langerhans [27]. Treatment with different doses of AE for 12 days showed significant improvement in serum insulin levels (Table 1), thus suggesting that the observed antihyperglycaemic effect of AE was due to its action at the pancreatic level by stimulating insulin secretion from viable β cells (insulin secretagogue) and/or triggering the regeneration of pancreatic β cells. Hence, the possible pancreatic effects of AE were further scrutinized through an immunohistochemical analysis of the pancreatic tissue. From the section presented in Figure 2, it could be concluded that almost all the insulin-positive areas in STZ-induced diabetic rats, treated and untreated, showed signs of degeneration and necrosis. Moreover, the areas of insulin-positive cells in these groups were significantly reduced as compared with normal rats. These conditions were presumed to coincide with a decrease in plasma insulin secretion and an increase in blood glucose levels. However, diabetic rats treated with AE (1000 and 500 mg/kg b.w.) showed a significant increase in serum insulin concentrations, which could be responsible for the improved blood glucose levels in these animals (Table 1). There are two possible explanations for this outcome. The bioactive compounds of AE might have acted by stimulating insulin secretion from viable β cells that were not destroyed during STZ induction, while compounds present in AE further enhanced the effects of circulating insulin [28,29]. These actions were not necessarily accompanied by any structural recovery in pancreatic β cells. This explains why there is no improvement in the insulin expression area of β cells pertaining to the regeneration effect of AE, as reflected in the immunohistochemical study of the pancreas. Several medicinal plants have been found to exert their hypoglycaemic activities via the stimulation of insulin secretion from pancreatic β cells. Examples are Aloe vera, *Gymnema sylvestre*, and *Hibiscus rosa-sinesis* [30,31,32].

To clarify the possible effect of AE in stimulating insulin secretion from viable β cells, the RIN-5F cell line was used. RIN-5F cell line is a cloned pancreatic β cell derived from rat tumour islet cells. This cell line provides a model for in vitro study of the biology of pancreatic islet cells, including the mechanisms that control insulin secretion [33,34]. As depicted in Figure 4, AE stimulated insulin secretion even in basal glucose (1.1 mM) conditions. A similar effect was seen in the glibenclamide-treated group. Thus, it could be hypothesized that AE’s insulin stimulatory effect was mediated, in part, by its ability to regulate insulin secretion from β cells. To test this hypothesis, a further study is required in order to pinpoint AE’s effects on the depolarization of the β cell membrane in terms of the opening and closure of ATP-sensitive K^+^ and/or voltage-gated Ca^2+^ channels; the expression of proinsulin mRNA; and extracellular Ca^2+^ influx [35]. All these factors could contribute to insulin exocytosis from β cells in the pancreas.

In addition to the insulin stimulatory effect, AE also produced a positive hepatoprotective effect. The liver plays a crucial role in glucose homeostasis. This role is due to its capability to control hepatic glucose production via two ways: The release of newly synthesized glucose in the blood stream during the fasting state; and the utilization and storage of glucose when glucose is elevated during the feeding state [36,37]. STZ administration causes substantial changes in the intracellular metabolism of most tissues, including the liver. Examples of such changes in the liver are fat deposition, the development of lesions, the congestion of portal vessels and sinusoids and the dilation of veins [38,39]. By using H&E staining, significant histopathological changes were observed in the liver tissue of DC rats as compared with NC (Figure 3). DC rats exhibited several of those aforementioned histopathological abnormalities. Treatment with AE significantly enhanced the lobular architecture and the central vein in livers that were earlier affected by STZ induction. It seems that AE protects hepatocytes from the toxic effects of STZ. This finding, though inconclusive, suggests that *Nypa fruticans* Wurmb.’s AE can act as a hepatoprotective agent. Similar findings were observed by Halima et al. [40] and Omar et al. [16] when studying the effect of apple cider vinegar on the livers of diabetic rats.

The present findings ought to be considered in the context of several limitations. Though our histoarchitectural results indicated a hepatoprotective effect, all liver function parameters should be measured to confirm it, including aspartate aminotransferase (AST), alanine aminotransferase (ALT), alkaline phosphatise (ALP), albumin and bilirubin and the level of antioxidants enzymes, namely superoxide dismutase (SOD), catalase (CAT) and glutathione (GST). Further studies with longer observational durations are needed in order to confirm our findings. This may be especially required as NPV is a dietary ingredient that is commonly ingested on daily basis in some rural communities; and a shorter treatment period had previously been shown to precipitate no significant improvement of diabetic blood glucose levels [10]. Furthermore, the cell culture technique used in the present work employed a basal level of glucose concentration. It is possible that observations made using different concentrations of glucose could, in the future, provide further insight into AE’s insulin-stimulatory effects.

## 5. Conclusions

In conclusion, these findings imply that constant consumption of NPV could bring positive effect on the pancreas and liver. Considering the close relationship of pancreas and liver with the progression of diabetes, NPV may provide a potential complementary alternative for the therapy of this metabolic disorder.

## Figures and Tables

**Table 1:** Effect of twice daily oral administration of different doses of AE for 12 days on blood glucose level, serum insulin level and body weight of STZ-induced diabetic rats.

Group	Blood Glucose (mmol/L)		Serum Insulin (μg/L)		Body Weight (g)	
	Day 0	Day 12	Day 0	Day 12	Day 0	Day 12
NC	4.5 ± 0.18	4.00 ± 0.20	0.42 ± 0.13	0.37 ± 0.01	216.33 ± 2.26	227.83 ± 1.35 ^$$^
DC	17.00 ± 0.67	19.97 ± 1.52 ^###^	0.21 ± 0.01	0.03 ± 0.02 ^###$$^	196 ± 5.07	168.5 ± 7.91 ^###$$^
Metformin	15.85 ± 1.24	8.40 ± 1.27 **^$^	0.21 ± 0.02	0.29 ± 0.01 ^###^**^$$^	209.17 ± 5.30	195.17 ± 9.37 ^#^
AE (1000 mg/kg b.w.)	16.95 ± 0.58	7.35 ± 0.70 ***^$$$^	0.19 ± 0.02	0.20 ± 0.03 ^##^*	203.83 ± 3.96	167.50 ± 8.80 ^###$$^
AE (500 mg/kg b.w.)	16.43 ± 0.93	8.35 ± 1.42 **^$$^	0.19 ± 0.02	0.25 ± 0.04 ^##^*^$^	206.5 ± 3.37	169.17 ± 4.44 ^###$$$^
AE (250 mg/kg b.w.)	15.47 ± 1.07	16.00 ± 2.86 ^###^	0.18 ± 0.03	0.18 ± 0.01 ^##^*	199.67 ± 3.51	166.67 ± 9.01 ^###$$^

Data are expressed as mean ± S.E.M., *n* = 6. * *p* < 0.05, ** *p* < 0.01, *** *p* < 0.001 versus DC. ^#^ *p* < 0.05, ^###^ *p* < 0.001 versus NC. ^$^ *p* < 0.05, ^$$^ *p* < 0.01, ^$$$^ *p* < 0.001 versus initial value (day 0). Streptozotocin, STZ. NC, normal control. DC, diabetic control. AE, aqueous extract. S.E.M, standard error of the mean. b.w., body weight.

**Table 2:** Effect of twice daily oral administration of different doses of AE for 12 days on the serum lipid profiles of normal and STZ-induced diabetic rats.

Treatment (mg/kg b.w.)	Serum Lipid Profile (mg/dL)				
	TC	TG	HDL	LDL	AIP
NC	64.68 ± 0.84	44.92 ± 3.20	39.83 ± 7.13	20.58 ± 1.26	0.05
DC	61.76 ± 4.17	69.03 ± 2.31 ^#^	36.58 ± 3.00	11.39 ± 1.63	0.28
Metformin (500 mg/kg b.w.)	62.84 ± 2.90	66.21 ± 5.90 ^#^	37.32 ± 4.22	12.28 ± 1.99	0.25
AE (1000 mg/kg b.w.)	65.62 ± 9.75	34.32 ± 3.78 **	29.2 ± 3.00	23.69 ± 7.06 **	0.07
AE (500 mg/kg b.w.)	37.70 ± 2.90 *^#^	28.34 ±3.00 **	22.53 ± 1.72 *^##^	6.86 ± 2.28	0.1
AE (250 mg/kg b.w.)	37.70 ±3.99 *^#^	38.75 ± 9.19 *	20.88 ±2.25 *^##^	8.99 ± 0.33	0.27

Data is expressed as mean ± S.E.M., *n* = 6. * *p* < 0.05, ** *p* < 0.01, *** *p* < 0.001 versus DC. ^#^ *p* < 0.05, ^###^ *p* < 0.001 versus NC. TC, total cholesterol. TG, triglycerides. HDL, high density lipoprotein. LDL, low density lipoprotein. AIP, atherogenic index of plasma. S.E.M, standard error of the mean.

## Abbreviations

The following abbreviations are used in this manuscript:

AE	Aqueous extract
NPV	*Nypa fruticans* Wurmb. vinegar
DM	Diabetes mellitus
STZ	Streptozotocin
b.w.	Body weight
i.p.	Intraperitoneal
p.o.	Per oral
SD	*Sprague-Dawley*
MTT	3-(4,5-dimethylthiazol-2-yl)-2,5-diphenyltetrazoliumbromide
NC	Normal control
DC	Diabetic control
